# Significance of the Glasgow prognostic score for short‐term surgical outcomes: A nationwide survey using the Japanese National Clinical Database

**DOI:** 10.1002/ags3.12456

**Published:** 2021-03-21

**Authors:** Yoshihiro Hiramatsu, Hiraku Kumamaru, Hirotoshi Kikuchi, Shiyori Usune, Kinji Kamiya, Hiroaki Miyata, Hiroyuki Konno, Yoshihiro Kakeji, Yuko Kitagawa, Hiroya Takeuchi

**Affiliations:** ^1^ The Japanese Society of Gastroenterological Surgery Minato‐ku Japan; ^2^ Department of Surgery Hamamatsu University School of Medicine Hamamatsu Japan; ^3^ Department of Perioperative Functioning Care and Support Hamamatsu University School of Medicine Hamamatsu Japan; ^4^ Department of Healthcare Quality Assessment Graduate School of Medicine University of Tokyo Bunkyo‐ku Japan; ^5^ Hamamatsu University School of Medicine Hamamatsu Japan; ^6^ Database Committee The Japanese Society of Gastroenterological Surgery Minato‐ku Japan

**Keywords:** gastrointestinal malignancies, gastrointestinal neoplasms, Japanese National Clinical Database, prognostic factors, surgical outcomes

## Abstract

**Aim:**

Preoperative inflammation‐based Glasgow prognostic score (GPS) is a useful tool for predicting long‐term prognosis in cancer patients. However, its association with postoperative short‐term outcomes remains unknown. The aim of this study is to investigate the association between GPS and postoperative morbidity and mortality among patients undergoing surgery for various gastrointestinal malignancies.

**Methods:**

Using the Japanese National Clinical Database, we analyzed the records of 312 357 patients with gastrointestinal malignancy who underwent six typical elective surgeries, including esophagectomy, distal gastrectomy, total gastrectomy, right hemicolectomy, low anterior resection, and pancreaticoduodenectomy, between January 2015 and December 2018. We assigned GPS of 0, 1, or 2 to patients with no, one, or both decreased albumin and elevated C‐reactive protein levels, respectively. We investigated the relationship of GPS with operative morbidity and mortality for each procedure with adjustments for patients' demographics, preoperative status, comorbidities, and cancer stages.

**Results:**

Crude operative morbidity was significantly higher for GPS 1 and 2 than GPS 0 patients in all procedures except pancreaticoduodenectomy. The postoperative length of hospital stay was significantly longer for GPS 1 and 2 patients in all procedures (*P* < .001). Operative mortality was also higher for GPS 1 and 2 patients in all procedures. The associations remained significant after adjustments for potential confounders of age, sex, physical status, tumor classification, use of preoperative therapy, and comorbidities.

**Conclusion:**

This nationwide study provides solid evidence on the strong association between GPS and postoperative outcomes.

## Graphical abstract

This study aimed to investigate the significance of Glasgow prognostic score (GPS) for operative morbidity and mortality in cancer patients. We analyzed records from the Japanese National Clinical Database. This study provides solid evidence to reinforce the significant association of GPS with operative morbidity and mortality.

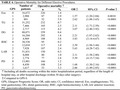

## 1 INTRODUCTION

The systemic inflammatory response has been associated with poor clinical outcomes in many cancer types.^1^, ^2^, ^3^ Elevated C‐reactive protein (CRP) concentration is the most common measure of the systemic inflammatory response in cancer patients due to its sensitivity, specificity, and reproducibility in hospital laboratories. Furthermore, the magnitude of the increase in CRP concentrations has been shown to be associated with poorer survival in cancer patients, independent of performance status, weight loss, tumor stage, and other high‐risk pathological features.^4^, ^5^, ^6^ A correlation between hypoalbuminemia and poor prognosis in patients with malignancies is also well‐recognized.^7^, ^8^ In cancer patients, malnutrition is often associated with anorexia‐cachexia syndrome, characterized by decreased food intake, hypoalbuminemia, weight loss, and muscle tissue wasting.^9^ Nutrition becomes the foremost concern as preoperative malnutrition is also an important risk factor for postoperative morbidity and mortality.^10^

Forrest et al developed the inflammation‐based Glasgow prognostic score (GPS) consisting of two simple components: serum levels of CRP and albumin.^11^ The GPS is one of the most useful indexes for predicting long‐term prognosis in various cancers.^12^, ^13^, ^14^, ^15^, ^16^, ^17^ However, the relationship between GPS and postoperative short‐term outcomes remains controversial.^18^, ^19^, ^20^, ^21^, ^22^ Preoperative serum levels of CRP and albumin have been reported as the risk factors for postoperative complications and mortality.^23^, ^24^, ^25^, ^26^, ^27^, ^28^, ^29^, ^30^, ^31^, ^32^ Despite advances in surgical techniques and devices, some patients who undergo the procedure experience clinically relevant postoperative complications, resulting in a protracted recovery period, delayed administration of adjuvant chemotherapy, and impaired quality of life.^33^, ^34^, ^35^, ^36^, ^37^, ^38^, ^39^ Therefore, risk management using a prediction tool based on preoperatively determining factors is important to obtain appropriate informed consent and perioperative management, as well as to minimize the medical cost burden.^40^

Several other predictive tools for cancer surgery prognosis have been developed, for example the prognostic nutritional index (PNI), the controlling nutritional status (CONUT) score, the CRP/Albumin ratio, and others.^20^, ^21^ These tools use a variety of factors that can be measured through standard clinical laboratory tests. However, since the Japanese National Clinical Database (NCD), from which data for this study were obtained, does not store data for factors such as total cholesterol, neutrophil count, and lymphocyte count, we excluded a number of these tools for use in this study. Furthermore, exact values for serum albumin and CRP levels were not available in the database for patients whose serum levels of these two markers fell within normal range. The modified GPS (mGPS), developed and used in various studies, does not show a clear superiority over the GPS for surgical outcome,^18^, ^20^, ^41^ and would have greatly reduced the number of score 1s in this study. We therefore elected to use the original GPS as the predictive tool to analyze in this study.

Based on previous reports,^18^, ^19^, ^20^, ^21^, ^22^, ^23^, ^24^, ^25^, ^26^, ^27^, ^28^, ^29^, ^30^, ^31^, ^32^ we hypothesized that the preoperative GPS could predict the postoperative short‐term outcomes as well as long‐term prognosis in various cancers. The present study aimed to investigate the significance of preoperative GPS in predicting postoperative morbidity and mortality in patients with gastrointestinal carcinoma. To our knowledge, this is the first study to demonstrate the impact of GPS on postoperative morbidity and mortality using extensive data from a nationwide database.

## 2 METHODS

### 2.1 The nationwide database system

The Japanese NCD, which started its data registration in 2011, has grown into a large nationwide database covering more than 95% of the surgeries performed by general surgeons in Japan.^39^ As of the end of December 2019, 5276 facilities have enrolled in the NCD, and about 1 500 000 cases are registered every year.^39^ The NCD is a nationwide project linked to the board certification system for surgery in Japan. The submission of cases to the NCD is a prerequisite for all member institutions of both the Japan Surgical Society and the Japanese Society of Gastrointestinal Surgery, and only registered cases can be used for board certification reviews. The data are evaluated annually using a web‐based data management system to ensure data traceability. The data are also validated against medical charts via audits to randomly selected institutions.^42^ The most recent laboratory data for each patient, obtained 90 days or less prior to surgery, are registered in the NCD.

The data from the NCD were provided to analysis teams after anonymization. Patients were provided with the opportunities to opt‐out from their clinical information being used for research. The approval by the Ethics Committee of Hamamatsu University School of Medicine was obtained before starting data analysis, and this study was registered at the UMIN Clinical Trials Registry as UMIN 000036761 (http://www.umin.ac.jp/ctr/index.htm).

### 2.2 Patients

We analyzed data from a total of 312 357 patients who underwent six typical procedures of gastroenterological surgery (esophagectomy [Eso], distal gastrectomy [DG], total gastrectomy [TG], right hemicolectomy [RHC], low anterior resection [LAR], and pancreaticoduodenectomy [PD]) between 1 January 2015 and 31 December 2018. We only included patients who underwent surgical procedures for malignancy (Eso for esophageal cancer; TG and DG for gastric cancer; RHC for colon cancer; LAR for rectal cancer; PD for pancreatic cancer). The exclusion criteria were as follows: (a) cases with distant metastasis or disseminated cancer; (b) emergent surgery cases; (c) cases with concomitant major surgery; (d) cases in which artificial respiration management was performed before surgery; (e) cases with coexisting preoperative sepsis, pneumonia, or open wounds; (f) patients who regularly used steroids before surgery; and (g) missing values for preoperative serum CRP and albumin (Table S1).

### 2.3 Assessment of GPS

We assessed patients' GPS as follows: patients with both elevated CRP (>10 mg/L) and hypoalbuminemia (<35 g/L) levels were allocated a score of 2; patients with only one of the biochemical abnormalities were allocated a score of 1; patients with neither of these abnormalities were allocated a score of 0.^11^

### 2.4 Study outcomes

We assessed the occurrence of postoperative complications categorized as grade III or above based on the Clavien‐Dindo (CD) classification,^43^ operative mortality, and the length of hospital stay after surgery. Postoperative complications included surgical site infection, wound dehiscence, anastomotic leakage, pancreatic fistula, bile leakage, pneumonia, unplanned intubation, pulmonary embolism, ventilator‐assisted respiration longer than 48 hours, progressive renal insufficiency, acute renal failure, urinary tract infection, cerebrovascular accident with neurological deficit, coma longer than 24 hours, peripheral nerve injury, cardiac arrest requiring cardiopulmonary resuscitation, myocardial infarction, bleeding complications defined by transfusions over one unit of blood, deep venous thrombosis, and sepsis. Detailed descriptions of the postoperative complications associated with individual cancers have been provided previously.^27^, ^28^, ^29^, ^30^, ^31^, ^32^, ^33^, ^34^, ^35^, ^36^, ^37^, ^38^, ^39^ Operative mortality was defined as death during the index hospitalization, regardless of the length of hospital stay, as well as death after hospital discharge in ≤30 days from the surgery date.

### 2.5 Statistical analysis

We tabulated the characteristics of the patients by GPS separately for each type of surgical procedure. We assessed the incidences of postoperative complications and operative mortality by GPS score for each procedure type and estimated the median and 10th‐90th percentiles for the length of postoperative hospital stay. We compared the postoperative length of stay across the three GPS groups using the Kruskal‐Wallis test. For each procedure type, relative odds for postoperative morbidity and mortality were estimated for GPS 1 and 2 groups as compared to GPS 0 group using multivariable logistic regression models, adjusting for patients' age; sex; American Society of Anesthesiologists‐Physical Status (ASA‐PS); T classification; N classification; use of preoperative chemo‐, radiation‐, or other therapy; and comorbidities including diabetes mellitus, hypertension, chronic obstructive pulmonary disease, cardiac disease, cerebrovascular disease, and kidney dysfunction. Tests were all two‐sided and *P‐*values <.05 were considered statistically significant. All statistical analyses were conducted using SAS 9.4 (SAS Institute).

## 3 RESULTS

The study population selection process for each procedure is summarized in Table S1. After subject selection, there were 20 541 Eso patients, 41 435 TG patients, 109 244 DG patients, 58 476 RHC patients, 62 693 LAR patients, and 19 968 PD patients.

### 3.1 GPS distribution

**FIGURE 1:**
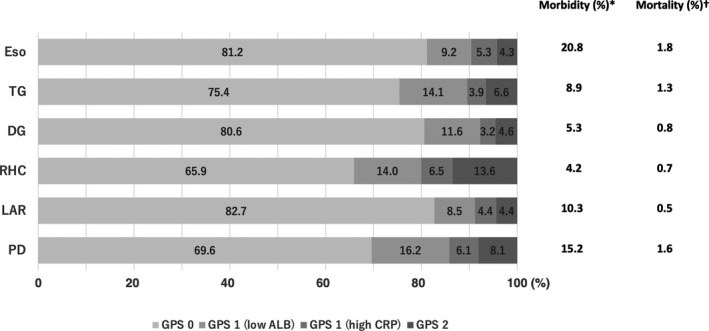
Glasgow Prognostic Score (GPS) distribution in the surgical procedures (%). *Operative morbidity included complications of grade 3 and greater according to Clavien‐Dindo classification. †Operative mortality included all deaths occurring within the index hospitalization period, regardless of the length of hospital stay, or after hospital discharge (within 30 d after surgery). Eso, esophagectomy; TG, total gastrectomy; DG, distal gastrectomy; RHC, right hemicolectomy; LAR, low anterior resection; PD, pancreaticoduodenectomy; ALB, albumin; CRP, C‐reactive protein

Figure 1 shows the GPS distribution of all patients whose data were analyzed, together with the overall incidence of postoperative mortality and morbidity (CD‐3 and above). Overall, 65.9%‐82.7% of the procedures were performed on patients with GPS 0. GPS distribution varied by the procedure type, but the proportion of patients with GPS 2 did not exceed 10% in any procedure except RHC, in which the proportion of patients with GPS 2 was 13.6%. The proportion of patients with GPS 2 was the smallest in Eso (4.3%), which had the highest operative morbidity rate among the six procedures examined. Despite high invasiveness and morbidity in PD, the proportion of patients with GPS 1 or 2 was over 30% and higher than other procedures similar to RHC, which is generally considered less invasive. We saw no clear association between the GPS distribution and procedure's overall incidence of operative morbidity and mortality. In the GPS 1 group, patients with lower albumin levels were predominant for all procedures.

Table 1 summarizes the demographic and comorbidity parameters of patients who underwent each procedure. The mean age of patients in all procedures was approximately 70 years (67.3‐73.7 years). Gender was disease‐specific and varied widely depending on each surgical procedure. Age, ASA‐PS, and clinical stage (cT, tumor invasion depth; and cN, lymph node metastasis) increased with an increase in GPS. Full tabulation of patients' characteristics, preoperative treatment, and comorbidities are presented in the supporting information material (Tables [Link], [Link], [Link], [Link], [Link], [Link]). In summary, patients with high GPS had higher frequencies of receiving preoperative treatment and had more preoperative comorbidities. Table S8 shows the breakdown of preoperative treatments administered to patients for the different procedure types.

**TABLE 1:** Clinical characteristics of study patients

	GPS	Number of patients	Age (y) (mean ± SD)	Sex (%) (Male ratio)	ASA‐PS (%)^a^	cT (%)^b^	cN (%)^c^
Eso		20 541	67.3 ± 8.9	82.0	8.4	59.5	52.7
	0	16 672	66.8 ± 8.9	81.6	7.3	55.4	50.7
	1	2988	68.7 ± 8.4	83.3	11.8	75.6	59.9
	2	881	70.5 ± 8.2	85.0	18.8	84.0	64.5
TG		41 435	70.5 ± 10.4	74.2	11.8	70.8	50.7
	0	31 252	69.2 ± 10.4	73.6	8.9	65.9	46.0
	1	7466	73.9 ± 9.3	74.8	19.6	84.1	63.5
	2	2717	75.4 ± 8.5	79.6	24.4	89.9	69.0
DG		109 244	70.6 ± 11.2	66.6	11.8	45.4	32.4
	0	88 071	69.2 ± 11.1	66.5	8.9	39.1	27.4
	1	16 204	75.8 ± 9.5	66.7	22.8	69.5	51.7
	2	4969	77.5 ± 8.8	67.8	28.8	79.4	57.4
RHC		58 476	73.7 ± 10.7	49.5	15.1	85.2	39.5
	0	38 538	72.2 ± 10.5	50.5	10.6	79.9	35.9
	1	12 010	76.3 ± 10.5	48.8	21.8	94.4	45.7
	2	7928	77.1 ± 10.5	45.5	26.5	97.2	47.8
LAR		62 693	67.3 ± 11.3	65.3	9.8	80.6	37.7
	0	51 831	66.4 ± 11.2	65.2	7.8	78.4	36.3
	1	8088	71.4 ± 10.7	65.9	18.4	89.9	44.2
	2	2774	72.3 ± 10.4	66.5	21.4	93.9	43.5
PD		19 968	69.5 ± 9.7	56.2	13.0	82.1	47.0
	0	13 901	68.7 ± 10.0	55.4	11.2	78.3	43.4
	1	4450	71.1 ± 8.7	58.2	16.4	90.2	54.3
	2	1617	72.3 ± 8.3	56.9	19.7	92.7	58.5

Abbreviations: ASA‐PS, American Society of Anesthesiologists‐Physical Status; cN, preoperative diagnosis of lymph node metastasis; cT, preoperative diagnosis of tumor invasion depth; DG, distal gastrectomy; Eso, esophagectomy; GPS, Glasgow Prognostic Score; LAR, low anterior resection; PD, pancreaticoduodenectomy; RHC, right hemicolectomy; SD, standard deviation; TG, total gastrectomy.

^a^ Represents proportion of patients with ASA‐PS class 3 and above.

^b^ Represents proportion of patients with stage T2 and above.

^c^ Represents proportion of patients with stage N1 and above.

### 3.2 Influence of GPS on operative morbidity and postoperative hospital stay

Table 2 shows the incidences of operative morbidity as well as the relative odds for operative morbidity from the logistic regression models for all procedures. The incidences of operative morbidity were higher for patients in higher GPS groups. The operative morbidity for patients with GPS 1 or GPS 2 was significantly higher than that for patients with GPS 0, for all procedures except PD. A remarkable difference was observed especially in the patients with GPS 2 in all procedures (odds ratio, 1.31‐1.68; *P* < .0001), except in PD (odds ratio, 1.15; *P* = .0577) from the multivariable logistic regression models. As shown in Tables [Link], [Link], [Link], [Link], [Link], [Link], the confidence intervals of odds ratios for GPS 1 and 2 patients had little or no overlap between these two groups in the operative morbidity for all procedures except in PD, suggesting significant difference. The odds ratios were higher for surgical procedures with relatively lower overall complication rates, such as DG, RHC, and LAR. On the contrary, the odds ratios were lower in those surgical procedures that had a higher incidence of postoperative morbidities, such as Eso and PD, and no significant difference was observed in PD. The full list of estimates from the multivariable regression analyses is presented in Tables [Link], [Link], [Link], [Link], [Link], [Link]. Together with GPS, poor physical health, advanced cancer stages as well as comorbidities such as past cerebrovascular or cardiovascular diseases, kidney dysfunction, and COPD were identified as strong risk predictors of complications after surgeries. The length of hospital stay after surgery also significantly increased with an increase in GPS in all procedures, including PD (*P* < .0001; Table 3). Table S15 shows the five most frequent postoperative complications in each surgical procedure.

**TABLE 2:** Operative morbidity for different elective procedures

	GPS	Number of patients	Operative morbidity^a^		OR^b^	95% CI	*P*‐value^b^
			n	%			
Eso	0	16 672	3312	19.9			
	1	2988	701	23.5	1.15	(1.04‐1.26)	.0049
	2	881	253	28.7	1.42	(1.22‐1.66)	<.0001
TG	0	31 252	2565	8.2			
	1	7466	779	10.4	1.11	(1.02‐1.22)	.0158
	2	2717	350	12.9	1.31	(1.16‐1.48)	<.0001
DG	0	88 071	4105	4.7			
	1	16 204	1233	7.6	1.26	(1.17‐1.35)	<.0001
	2	4969	468	9.4	1.43	(1.29‐1.60)	<.0001
RHC	0	38 538	1324	3.4			
	1	12 010	596	5.0	1.27	(1.15‐1.41)	<.0001
	2	7928	529	6.7	1.68	(1.50‐1.88)	<.0001
LAR	0	51 831	4968	9.6			
	1	8088	1057	13.1	1.32	(1.22‐1.42)	<.0001
	2	2774	433	15.6	1.58	(1.42‐1.77)	<.0001
PD	0	13 901	2063	14.8			
	1	4450	697	15.7	1.06	(0.96‐1.16)	.2624
	2	1617	275	17.0	1.15	(0.99‐1.32)	.0577

Abbreviations: CI, confidence interval; DG, distal gastrectomy; Eso, esophagectomy; GPS, Glasgow Prognostic Score; LAR, low anterior resection; OR, odds ratio; PD, pancreaticoduodenectomy; RHC, right hemicolectomy; TG, total gastrectomy.

^a^ Including grade 3 and grater according to Clavien‐Dindo classification.

^b^ Compared to GPS 0.

**TABLE 3:** Postoperative length of hospital stay

	GPS	Postoperative hospital stay (d) median (10th‐90th percentiles)	*P*‐value
Eso	0	14 (23‐62)	<.0001
	1	15 (26‐79)	
	2	15 (28‐90)	
TG	0	15 (9‐36)	<.0001
	1	17 (10‐49)	
	2	20 (11‐57)	
DG	0	12 (8‐27)	<.0001
	1	15 (9‐42)	
	2	18 (10‐56)	
RHC	0	11 (7‐22)	<.0001
	1	14 (8‐33)	
	2	15 (8‐43)	
LAR	0	14 (8‐35)	<.0001
	1	17 (9‐48)	
	2	20 (10‐55)	
PD	0	24 (14‐51)	<.0001
	1	26 (14‐56)	
	2	26 (14‐59)	

Abbreviations: DG, distal gastrectomy; Eso, esophagectomy; GPS, Glasgow Prognostic Score; LAR, low anterior resection; PD, pancreaticoduodenectomy; RHC, right hemicolectomy; TG, total gastrectomy.

### 3.3 Influence of GPS on operative mortality

The crude operative mortality by GPS groups as well as the relative odds for postoperative death for GPS 1 and 2 vs 0 are shown in Table 4. There was almost no overlap in confidence intervals for odds ratios of operative mortality between GPS 1 and 2, and the higher GPS score was associated with higher operative mortality for all procedures (Table 4 and [Link], [Link], [Link], [Link], [Link], [Link]). The association between GPS and the incidence of operative mortality was more pronounced than that between GPS and the incidence of operative morbidity, and it was more noticeable for patients with GPS 2 (Eso, 2.62; TG, 3.07; DG, 3.83; RHC, 5.63; LAR, 5.59; PD, 2.06; *P* < .0001). Similar to the analysis of operative morbidity, the odds ratio for mortality was higher in the high GPS groups among patients undergoing procedures that had low mortality such as TG, DG, RHC, and LAR. In these four procedure types, with crude mortality of ≤0.7% for patients with GPS 0, the mortality of GPS 2 patients was more than three times higher. Associations between factors such as age, advanced cancer stage, and comorbidities with mortality after surgeries were stronger compared with those with incidences of post‐surgical complications (Tables [Link], [Link], [Link], [Link], [Link], [Link]).

**TABLE 4:** Operative mortality for different elective procedures

	GPS	Number of patients	Operative mortality^a^		OR^b^	95% CI	*P‐*value^b^
			n	%			
Eso	0	16 672	226	1.4			
	1	2988	87	2.9	1.57	(1.20‐2.04)	.0010
	2	881	52	5.9	2.62	(1.88‐3.65)	<.0001
TG	0	31 252	232	0.7			
	1	7466	185	2.5	2.11	(1.71‐2.59)	<.0001
	2	2717	115	4.2	3.07	(2.40‐3.92)	<.0001
DG	0	88 071	359	0.4			
	1	16 204	292	1.8	2.21	(1.87‐2.62)	<.0001
	2	4969	188	3.8	3.83	(3.14‐4.67)	<.0001
RHC	0	38 538	95	0.2			
	1	12 010	117	1.0	2.59	(1.94‐3.44)	<.0001
	2	7928	187	2.4	5.63	(4.28‐7.42)	<.0001
LAR	0	51 831	150	0.3			
	1	8088	80	1.0	2.11	(1.58‐2.80)	<.0001
	2	2774	77	2.8	5.59	(4.15‐7.54)	<.0001
PD	0	13 901	166	1.2			
	1	4450	108	2.4	1.68	(1.30‐2.16)	<.0001
	2	1617	53	3.3	2.06	(1.49‐2.85)	<.0001

Abbreviations: CI, confidence interval; DG, distal gastrectomy; Eso, esophagectomy; GPS, Glasgow Prognostic Score; LAR, low anterior resection; OR, odds ratio; PD, pancreaticoduodenectomy; RHC, right hemicolectomy; TG, total gastrectomy.

^a^ Including all deaths occurring within the index hospitalization period, regardless of the length of hospital stay, or after hospital discharge (within 30 d after surgery).

^b^ Compared to GPS 0.

## 4 DISCUSSION

We conducted a large‐scale study on the association between GPS and short‐term operative outcomes in gastroenterological surgery, using data accumulated in the NCD in Japan. This nationwide survey confirmed that GPS is a strong risk predictor of operative morbidity and mortality and has a similar impact across different procedures in the field of gastrointestinal surgery.

Several previous studies have investigated the causal relationship between GPS and short‐term surgical outcomes, such as morbidity and mortality after surgery in various fields, including gastroenterology.^18^, ^19^, ^20^, ^21^, ^22^, ^25^ However, according to the literature, the effects of GPS on postoperative morbidity and mortality vary among operative procedures and even for the same procedure, so the influence of GPS on operative short‐term outcomes was controversial. In addition, while the sample size in the previous reports had been limited to approximately 1000 patients, the number of patients in this study was much greater than the previous studies, allowing us to separately evaluate data from different gastroenterological procedures at once.

Operative morbidity was significantly higher in patients with higher GPS for all procedures except PD. Interestingly, the procedures associated with the highest increase in operative morbidity were DG and RHC, which are generally considered to be less invasive and twice as safe as other procedures. On the contrary, Eso and PD, which had high morbidity in patients with GPS 0, had a smaller impact on the association of GPS with morbidity, and no significant difference was observed in PD. In a highly invasive surgery with high operative morbidity, it may be considered that patients with poor preoperative conditions were not indicated and appropriate alternative treatment was selected. It is also possible that PD has variables related to its morbidity that are more significant than GPS. Thus, as a risk factor for postoperative complications in PD, not only the preoperative condition of the patient, but also the surgical procedure itself may have a large impact. However, it was shown that the postoperative hospital stay was prolonged as the GPS increased in all surgical procedures including PD. In this study, we defined operative morbidity as grade III or higher complications of CD classification; however, GPS might have an effect on the incidence of postoperative complications less than grade III. The results of the previous studies were conflicting about the effect of GPS on operative morbidity, but those of the present study allowed us to conclude that high morbidity is predicted for patients with high GPS for almost all the procedures evaluated.

Operative mortality was also significantly increased in patients with higher GPS for all procedures. The highest increase in operative mortality, i.e. by 5.63 of the odds ratio, was observed for RHC, while that for PD increased by 2.06 of the odds ratio. The impact of GPS on mortality was greater than the impact on morbidity in all surgical procedures evaluated, even in patients with GPS 1. Interestingly, the influence of GPS on operative mortality was also greater in procedures which are generally considered to be less invasive and relatively safe, such as DG, RHC, and LAR, than the influence in the procedures which had high morbidity for patients with GPS 0, such as Eso and PD. Therefore, it is suggested that an appropriate preoperative risk assessment is important even for less invasive procedures, and in some cases modified surgery, multistep surgery, or alternative therapy needs to be considered. Previous studies that have evaluated the influence of GPS on short‐term postoperative outcomes have a small sample size,^18^, ^19^, ^20^, ^21^, ^22^, ^25^ and few have investigated the relationship between GPS and operative mortality.^19^, ^22^ The results of this study indicate that the GPS can predict the risk of short‐term postoperative mortality.

McMillan et al developed the mGPS and demonstrated that it was significantly associated with overall and cancer‐specific survival for patients with colorectal cancer.^41^ However, some studies showed that the GPS reflected the prognosis of patients with curable gastric cancer more accurately than the mGPS.^18^, ^20^ In these studies, the number of patients with hypoalbuminemia in the absence of an elevated CRP concentration who changed from GPS 1 to mGPS 0 was different, with the former being 16/109 (14.7%) and the latter being 52/92 (56.5%). Compared to other preoperative evaluation indicators such as PNI and CONUT, GPS tends to increase the number of patients in the normal group (GPS 0). Using mGPS, the number of patients with a 0 score is even higher than the number of patients using the GPS. In this study, the number of patients with lower albumin levels was predominant in the GPS 1 group, so the percentage of the normal group in the mGPS analysis was even greater.

While GPS is recognized as one of the most useful indicators for predicting long‐term prognosis in various cancers,^12^, ^13^, ^14^, ^15^, ^16^, ^17^ its usefulness as a predictor in the short‐term outcomes of patients undergoing surgery remains unclear.^18^, ^19^, ^20^, ^21^, ^22^, ^25^ Our multivariable analysis revealed that while the common risk factors of perioperative morbidity or mortality, including age, gender, ASA‐PS, tumor depth, lymph node metastasis, and preoperative comorbidity, were consistently valuable predicting factors, the GPS was also an independent risk predictor of operative morbidity and mortality in patients who underwent surgical treatment for gastroenterological malignancies. Since the GPS has the advantage of being easy to obtain prior to surgical intervention, it may be a useful tool for routine evaluation during gastrointestinal cancer treatment planning. The GPS provides a preoperative means to predict short‐term postoperative outcomes. Postoperative complications have been reported to worsen long‐term prognosis after surgery,^44^ and their incidence has been reportedly reduced by the intervention of the multidisciplinary perioperative management team.^45^ Thus, it is important to pay attention to perioperative care for patients with an elevated GPS for the reduction of postoperative complications and improvement of long‐term prognosis. Some research has reported that exercise and/or nutrition prehabilitation is associated with positive effects for weight maintenance and surgical complications.^46^, ^47^ However, a recent systematic review demonstrated the lack of strong evidence to determine the most optimal methods of preoperative support for patients undergoing gastrointestinal cancer resection.^46^, ^47^ There is also no evidence that an improvement of GPS prior to surgery will result in better short‐term outcomes in gastroenterological surgeries. It would be important and interesting for a future investigation to assess the optimal protocol for preoperative intervention and clarify whether a preoperative improvement of GPS correlates with an improved surgical outcome.

This study has the advantage of involving a large number of patients nationwide. However, there are limitations due to its observational design. While we adjusted for various risk factors for postoperative complications and mortality, there may be residual confounding from factors not included in the model. Certain details such as tumor location, surgery time, intraoperative bleeding, and the extent of lymphadenectomy were not assessed. In addition, the outcomes obtained in this study were influenced by various factors including hospital volume, training status and compliance, surgical specialization, resource utilization, and procedure‐specific variables, which may influence the clinical outcomes of the surgical procedures. However, our study highlights the correlation between the GPS and operative morbidity and mortality, and the GPS is expected to be a useful tool to preoperatively predict the short‐term outcomes of various gastroenterological surgeries.

In conclusion, we investigated the relationship of GPS with clinical outcomes of several gastroenterological surgical procedures based on data from 312 357 patients obtained from a Japanese nationwide database. This study demonstrated the significant and strong association between GPS and operative morbidity, as well as operative mortality for all surgical procedures investigated. This Japanese nationwide study provides novel evidence that the GPS is a significant risk predictor for short‐term outcomes after gastrointestinal surgery.

## DISCLOSURE

Ethical Statement: The protocol for this research project has been approved by the Ethics Committee of Hamamatsu University School of Medicine and it conforms to the provisions of the Declaration of Helsinki. This study was registered at the UMIN Clinical Trials Registry as UMIN 000036761 (http://www.umin.ac.jp/ctr/index.htm).

Conflicts of Interest and Source of Funding: HK, SU, and HM are affiliated with the Department of Healthcare Quality Assessment at the University of Tokyo, which is a social collaboration program supported by the National Clinical Database, Johnson & Johnson KK, and Nipro Corporation. For the remaining authors, none were declared.

Author Contributions: All listed authors have contributed significantly to, and are in agreement with, the content of the manuscript. Each author's contribution is as follows. Study conception and design: Yoshihiro Hiramatsu, Hiroyuki Konno, and Hiroya Takeuchi. Acquisition of data: Yoshihiro Kakeji and Yuko Kitagawa. Analysis and interpretation of data: Yoshihiro Hiramatsu, Hiraku Kumamaru, Hirotoshi Kikuchi, Shiyori Usune, Kinji Kamiya, Hiroaki Miyata and Hiroya Takeuchi. Participation in drafting the article or revising it critically for important intellectual content: All authors. Final approval of the version to be published: All authors.

## Supporting information

Table S1

Table S2

Table S3

Table S4

Table S5

Table S6

Table S7

Table S8

Table S9

Table S10

Table S11

Table S12

Table S13

Table S14

Table S15
