# Supplementary material for: Significance of the Glasgow prognostic score for short‐term surgical outcomes: A nationwide survey using the Japanese National Clinical Database
Source: Ann Gastroenterol Surg. 2021 Mar 21;5(5):659–68. doi: 10.1002/ags3.12456 (PMC8452482; doi:10.1002/ags3.12456)
Supplement: Supplementary file 1 — Table S1 [file AGS3-5-659-s009.docx]

| **Table S1.** Study Population Selection Process | | | | | | |
| --- | --- | --- | --- | --- | --- | --- |
| **Selection criteria** | **Eso** | **TG** | **DG** | **RHC** | **LAR** | **PD** |
| Number of patients undergoing each procedure | 24,409 | 61,530 | 143,992 | 90,837 | 85,401 | 44,812 |
| Malignant disease† | 23,370 | 58,752 | 136,127 | 81,151 | 79,974 | 22,643 |
| Without distant metastasis or dissemination | 22,578 | 51,915 | 128,502 | 70,638 | 72,008 | 21,885 |
| Elective surgery | 22,503 | 51,466 | 127,636 | 67,828 | 71,434 | 21,806 |
| Without associated surgeries simultaneously | 21,861 | 46,490 | 122,616 | 65,698 | 70,309 | 21,234 |
| Without artificial respiratory management | 21,841 | 46,470 | 122,573 | 65,674 | 70,284 | 21,227 |
| Without sepsis, pneumonia, and open wounds | 21,672 | 46,299 | 122,107 | 65,289 | 70,027 | 21,112 |
| Without regularly steroid use | 21,454 | 45,862 | 120,777 | 64,592 | 69,509 | 20,841 |
| Available data of preoperative CRP or albumin | 20,541 | 41,435 | 109,244 | 58,476 | 62,693 | 19,968 |
| † For PD, only pancreatic cancers were selected and non-pancreatic cancers were excluded.  Eso, esophagectomy; TG, total gastrectomy; DG, distal gastrectomy; RHC, right hemicolectomy; LAR, low anterior resection; PD, pancreaticoduodenectomy; CRP, C-reactive protein concentration. | | | | | | |
