# Supplementary material for: Significance of the Glasgow prognostic score for short‐term surgical outcomes: A nationwide survey using the Japanese National Clinical Database
Source: Ann Gastroenterol Surg. 2021 Mar 21;5(5):659–68. doi: 10.1002/ags3.12456 (PMC8452482; doi:10.1002/ags3.12456)
Supplement: Supplementary file 2 — Table S2 [file AGS3-5-659-s008.docx]

| **Table S2.** Background Parameters of Patients with Esophagectomy | | | | | | | | | | | |
| --- | --- | --- | --- | --- | --- | --- | --- | --- | --- | --- | --- |
|  | |  |  | **GPS** | | | | | | | |
|  | |  |  | **0 (n=16,672)** | |  | **1 (n=2,988)** | |  | **2 (n=881)** | |
| **Characteristics** | | |  | **n** | **%** |  | **n** | **%** |  | **n** | **%** |
| Age (years) | | <60 |  | 3,095 | 18.6 |  | 393 | 13.2 |  | 80 | 9.1 |
|  | | <70 |  | 6,769 | 40.6 |  | 1,115 | 37.3 |  | 315 | 35.8 |
|  | | <80 |  | 6,012 | 36.1 |  | 1,260 | 42.2 |  | 376 | 42.7 |
|  | | 80≤ |  | 796 | 4.8 |  | 220 | 7.4 |  | 110 | 12.5 |
| Sex | | Male |  | 13,597 | 81.6 |  | 2,489 | 83.3 |  | 749 | 85.0 |
|  | | Female |  | 3,075 | 18.4 |  | 499 | 16.7 |  | 132 | 15.0 |
| ASA-PS | | 1 |  | 3,441 | 20.6 |  | 436 | 14.6 |  | 93 | 10.6 |
|  | | 2 |  | 12,017 | 72.1 |  | 2,200 | 73.6 |  | 622 | 70.6 |
|  | | 3 |  | 11,90 | 7.1 |  | 346 | 11.6 |  | 161 | 18.3 |
|  | | 4 |  | 11 | 0.1 |  | 5 | 0.2 |  | 4 | 0.5 |
|  | | 5 |  | 13 | 0.1 |  | 1 | 0.0 |  | 1 | 0.1 |
| cT | | T0 |  | 300 | 1.8 |  | 66 | 2.2 |  | 18 | 2.0 |
|  | | Tis |  | 53 | 0.3 |  | 4 | 0.1 |  | 2 | 0.2 |
|  | | T1 |  | 7,012 | 42.1 |  | 641 | 21.5 |  | 112 | 12.7 |
|  | | T2 |  | 2,500 | 15.0 |  | 397 | 13.3 |  | 86 | 9.8 |
|  | | T3 |  | 5,964 | 35.8 |  | 1,518 | 50.8 |  | 523 | 59.4 |
|  | | T4 |  | 768 | 4.6 |  | 343 | 11.5 |  | 131 | 14.9 |
|  | | TX |  | 75 | 0.4 |  | 19 | 0.6 |  | 9 | 1.0 |
| cN | | N0 |  | 8,163 | 49.0 |  | 1,176 | 39.4 |  | 306 | 34.7 |
|  | | N1 |  | 4,582 | 27.5 |  | 881 | 29.5 |  | 262 | 29.7 |
|  | | N2 |  | 2,844 | 17.1 |  | 660 | 22.1 |  | 225 | 25.5 |
|  | | N3 |  | 1,035 | 6.2 |  | 249 | 8.3 |  | 81 | 9.2 |
|  | | NX |  | 48 | 0.3 |  | 22 | 0.7 |  | 7 | 0.8 |
| Preoperative treatment | | |  | 8,834 | 53.0 |  | 1,926 | 64.5 |  | 545 | 61.9 |
| Preoperative comorbidity | | | |  |  |  |  |  |  |  |  |
|  | Diabetes mellitus | |  | 2,398 | 14.4 |  | 483 | 16.2 |  | 353 | 17.5 |
|  | Hypertension | |  | 6,420 | 38.5 |  | 1,147 | 38.4 |  | 86 | 40.1 |
|  | COPD | |  | 1,304 | 7.8 |  | 250 | 8.4 |  | 55 | 9.8 |
|  | Cardiac disease | |  | 510 | 3.1 |  | 122 | 4.1 |  | 37 | 6.2 |
|  | Cerebrovascular disease | | | 453 | 2.7 |  | 106 | 3.5 |  | 7 | 4.2 |
|  | Kidney dysfunction | |  | 34 | 0.20 |  | 26 | 0.9 |  | 80 | 0.8 |
| GPS, Glasgow prognostic score; ASA-PS, American Society of Anesthesiologists - Physical Status; cT, preoperative diagnosis of tumor invasion depth; cN, preoperative diagnosis of lymph node metastasis; COPD, chronic obstructive pulmonary disease. | | | | | | | | | | | |
