# Supplementary material for: Significance of the Glasgow prognostic score for short‐term surgical outcomes: A nationwide survey using the Japanese National Clinical Database
Source: Ann Gastroenterol Surg. 2021 Mar 21;5(5):659–68. doi: 10.1002/ags3.12456 (PMC8452482; doi:10.1002/ags3.12456)
Supplement: Supplementary file 3 — Table S3 [file AGS3-5-659-s003.docx]

| **Table S3.** Background Parameters of Patients with Total Gastrectomy | | | | | | | | | | | |
| --- | --- | --- | --- | --- | --- | --- | --- | --- | --- | --- | --- |
|  | |  |  | **GPS** | | | | | | | |
|  | |  |  | **0 (n=31,252)** | |  | **1 (n=7,466)** | |  | **2 (n=2,717)** | |
| **Characteristics** | | |  | **n** | **%** |  | **n** | **%** |  | **n** | **%** |
| Age (years) | | <60 |  | 4,681 | 15.0 |  | 469 | 6.3 |  | 109 | 4.0 |
|  | | <70 |  | 9,934 | 31.8 |  | 1,701 | 22.8 |  | 545 | 20.1 |
|  | | <80 |  | 11,969 | 38.3 |  | 3,063 | 41.0 |  | 1,113 | 41.0 |
|  | | 80≤ |  | 4,668 | 14.9 |  | 2,233 | 29.9 |  | 950 | 35.0 |
| Sex | | Male |  | 23,014 | 73.6 |  | 5,585 | 74.8 |  | 2,162 | 79.6 |
|  | | Female |  | 8,238 | 26.4 |  | 1,881 | 25.2 |  | 555 | 20.4 |
| ASA-PS | | 1 |  | 7,331 | 23.5 |  | 916 | 12.3 |  | 268 | 9.9 |
|  | | 2 |  | 21,146 | 67.7 |  | 5,084 | 68.1 |  | 1,787 | 66.8 |
|  | | 3 |  | 2,730 | 8.7 |  | 1,429 | 19.1 |  | 644 | 23.7 |
|  | | 4 |  | 35 | 0.1 |  | 34 | 0.5 |  | 18 | 0.7 |
|  | | 5 |  | 10 | 0.0 |  | 3 | 0.0 |  | 0 | 0.0 |
| cT | | T0 |  | 85 | 0.3 |  | 21 | 0.3 |  | 1 | 0.0 |
|  | | Tis |  | 114 | 0.4 |  | 11 | 0.1 |  | 6 | 0.2 |
|  | | T1 |  | 10,396 | 33.3 |  | 1,140 | 15.3 |  | 262 | 9.6 |
|  | | T2 |  | 4,544 | 14.5 |  | 749 | 10.0 |  | 205 | 7.5 |
|  | | T3 |  | 7,920 | 25.3 |  | 2,372 | 31.8 |  | 879 | 32.4 |
|  | | T4 |  | 8,135 | 26.0 |  | 3,159 | 42.3 |  | 1,358 | 50.0 |
|  | | TX |  | 58 | 0.2 |  | 14 | 0.2 |  | 6 | 0.2 |
| cN | | N0 |  | 16,815 | 53.8 |  | 2,700 | 36.2 |  | 820 | 30.2 |
|  | | N1 |  | 5,200 | 16.6 |  | 1,380 | 18.5 |  | 525 | 19.3 |
|  | | N2 |  | 4,580 | 14.7 |  | 1,476 | 19.8 |  | 628 | 23.1 |
|  | | N3 |  | 4,596 | 14.7 |  | 1,883 | 25.2 |  | 723 | 26.6 |
|  | | NX |  | 61 | 0.2 |  | 27 | 0.4 |  | 21 | 0.8 |
| Preoperative treatment | | |  | 3,611 | 11.6 |  | 1,203 | 16.1 |  | 418 | 15.4 |
| Preoperative comorbidity | | | |  |  |  |  |  |  |  |  |
|  | Diabetes mellitus | |  | 5,706 | 18.3 |  | 1,625 | 21.8 |  | 624 | 23.0 |
|  | Hypertension | |  | 12,484 | 39.9 |  | 3,356 | 45.0 |  | 1268 | 46.7 |
|  | COPD | |  | 1,567 | 5.0 |  | 479 | 6.4 |  | 191 | 7.0 |
|  | Cardiac disease | |  | 1,457 | 4.7 |  | 561 | 7.5 |  | 228 | 8.4 |
|  | Cerebrovascular disease | | | 1,070 | 3.4 |  | 470 | 6.3 |  | 211 | 7.8 |
|  | Kidney dysfunction | |  | 102 | 3.8 |  | 115 | 4.2 |  | 54 | 2.0 |
| GPS, Glasgow prognostic score; ASA-PS, American Society of Anesthesiologists - Physical Status; cT, preoperative diagnosis of tumor invasion depth; cN, preoperative diagnosis of lymph node metastasis; COPD, chronic obstructive pulmonary disease. | | | | | | | | | | | |
