# Supplementary material for: Significance of the Glasgow prognostic score for short‐term surgical outcomes: A nationwide survey using the Japanese National Clinical Database
Source: Ann Gastroenterol Surg. 2021 Mar 21;5(5):659–68. doi: 10.1002/ags3.12456 (PMC8452482; doi:10.1002/ags3.12456)
Supplement: Supplementary file 4 — Table S4 [file AGS3-5-659-s007.docx]

| **Table S4.** Background Parameters of Patients with Distal Gastrectomy | | | | | | | | | | | |
| --- | --- | --- | --- | --- | --- | --- | --- | --- | --- | --- | --- |
|  | |  |  | **GPS** | | | | | | | |
|  | |  |  | **0 (n=88,071)** | |  | **1 (n=16,204)** | |  | **2 (n=4,969)** | |
| **Characteristics** | | |  | **n** | **%** |  | **n** | **%** |  | **n** | **%** |
| Age (years) | | <60 |  | 14,619 | 16.6 |  | 807 | 5.0 |  | 147 | 3.0 |
|  | | <70 |  | 27,017 | 30.7 |  | 3,030 | 18.7 |  | 773 | 15.6 |
|  | | <80 |  | 31,320 | 35.6 |  | 6,042 | 37.3 |  | 1,749 | 35.2 |
|  | | 80≤ |  | 15,115 | 17.2 |  | 6,325 | 39.0 |  | 2,300 | 46.3 |
| Sex | | Male |  | 58,528 | 66.5 |  | 10,810 | 66.7 |  | 3,367 | 67.8 |
|  | | Female |  | 29,543 | 33.5 |  | 5,394 | 33.3 |  | 1,602 | 32.2 |
| ASA-PS | | 1 |  | 22,370 | 25.4 |  | 1,963 | 12.1 |  | 433 | 8.7 |
|  | | 2 |  | 57,900 | 65.7 |  | 10,547 | 65.1 |  | 3,106 | 62.5 |
|  | | 3 |  | 7,688 | 8.7 |  | 3,620 | 22.3 |  | 1,392 | 28.0 |
|  | | 4 |  | 83 | 0.1 |  | 68 | 0.4 |  | 38 | 0.8 |
|  | | 5 |  | 30 | 0.0 |  | 6 | 0.0 |  | 0 | 0.0 |
| cT | | T0 |  | 272 | 0.3 |  | 36 | 0.2 |  | 9 | 0.2 |
|  | | Tis |  | 493 | 0.6 |  | 40 | 0.2 |  | 8 | 0.2 |
|  | | T1 |  | 52,703 | 59.8 |  | 4,835 | 29.8 |  | 997 | 20.1 |
|  | | T2 |  | 12,497 | 14.2 |  | 2,120 | 13.1 |  | 535 | 10.8 |
|  | | T3 |  | 12,711 | 14.4 |  | 4,268 | 26.3 |  | 1,507 | 30.3 |
|  | | T4 |  | 9,225 | 10.5 |  | 4,879 | 30.1 |  | 1,902 | 38.3 |
|  | | TX |  | 170 | 0.2 |  | 26 | 0.2 |  | 11 | 0.2 |
| cN | | N0 |  | 63,743 | 72.4 |  | 7,735 | 47.7 |  | 2,069 | 41.6 |
|  | | N1 |  | 11,115 | 12.6 |  | 2,948 | 18.2 |  | 928 | 18.7 |
|  | | N2 |  | 7,675 | 8.7 |  | 2,724 | 16.8 |  | 991 | 19.9 |
|  | | N3 |  | 5,376 | 6.1 |  | 2,711 | 16.7 |  | 933 | 18.8 |
|  | | NX |  | 162 | 0.2 |  | 86 | 0.5 |  | 48 | 1.0 |
| Preoperative treatment | | |  | 7,726 | 8.8 |  | 1,951 | 12.0 |  | 619 | 12.5 |
| Preoperative comorbidity | | | |  |  |  |  |  |  |  |  |
|  | Diabetes mellitus | |  | 15,836 | 18.0 |  | 3,616 | 22.3 |  | 1,085 | 21.8 |
|  | Hypertension | |  | 35,598 | 40.4 |  | 7,650 | 47.2 |  | 2,401 | 48.3 |
|  | COPD | |  | 4,144 | 4.7 |  | 1,001 | 6.2 |  | 302 | 6.1 |
|  | Cardiac disease | |  | 4,075 | 4.6 |  | 1,342 | 8.3 |  | 519 | 10.4 |
|  | Cerebrovascular disease | | | 2,889 | 3.3 |  | 1,095 | 6.8 |  | 386 | 7.8 |
|  | Kidney dysfunction | |  | 412 | 0.5 |  | 322 | 2.0 |  | 127 | 2.6 |
| GPS, Glasgow prognostic score; ASA-PS, American Society of Anesthesiologists - Physical Status; cT, preoperative diagnosis of tumor invasion depth; cN, preoperative diagnosis of lymph node metastasis; COPD, chronic obstructive pulmonary disease. | | | | | | | | | | | |
