# Supplementary material for: Significance of the Glasgow prognostic score for short‐term surgical outcomes: A nationwide survey using the Japanese National Clinical Database
Source: Ann Gastroenterol Surg. 2021 Mar 21;5(5):659–68. doi: 10.1002/ags3.12456 (PMC8452482; doi:10.1002/ags3.12456)
Supplement: Supplementary file 5 — Table S5 [file AGS3-5-659-s002.docx]

| **Table S5.** Background Parameters of Patients with Right Hemicolectomy | | | | | | | | | | | |
| --- | --- | --- | --- | --- | --- | --- | --- | --- | --- | --- | --- |
|  | |  |  | **GPS** | | | | | | | |
|  | |  |  | **0 (n=38,538)** | |  | **1 (n=12,010)** | |  | **2 (n=7,928)** | |
| **Characteristics** | | |  | **n** | **%** |  | **n** | **%** |  | **n** | **%** |
| Age (years) | | <60 |  | 4,078 | 10.6 |  | 740 | 6.2 |  | 419 | 5.3 |
|  | | <70 |  | 9,495 | 24.6 |  | 2,030 | 16.9 |  | 1,265 | 16.0 |
|  | | <80 |  | 15,202 | 39.4 |  | 4,074 | 33.9 |  | 2,531 | 31.9 |
|  | | 80≤ |  | 9,763 | 25.3 |  | 5,166 | 43.0 |  | 3,713 | 46.8 |
| Sex | | Male |  | 19,459 | 50.5 |  | 5,864 | 48.8 |  | 3,610 | 45.5 |
|  | | Female |  | 19,079 | 49.5 |  | 6,146 | 51.2 |  | 4,318 | 54.5 |
| ASA-PS | | 1 |  | 8,575 | 22.3 |  | 1,573 | 13.1 |  | 792 | 10.0 |
|  | | 2 |  | 25,861 | 67.1 |  | 7,814 | 65.1 |  | 5,036 | 63.5 |
|  | | 3 |  | 4,041 | 10.5 |  | 2,564 | 21.3 |  | 2,031 | 25.6 |
|  | | 4 |  | 43 | 0.1 |  | 53 | 0.4 |  | 62 | 0.8 |
|  | | 5 |  | 18 | 0.0 |  | 6 | 0.0 |  | 7 | 0.1 |
| cT | | T0 |  | 110 | 0.3 |  | 8 | 0.1 |  | 9 | 0.1 |
|  | | Tis |  | 1,466 | 3.8 |  | 165 | 1.4 |  | 54 | 0.7 |
|  | | T1 |  | 6,109 | 15.9 |  | 491 | 4.1 |  | 146 | 1.8 |
|  | | T2 |  | 5,699 | 14.8 |  | 782 | 6.5 |  | 258 | 3.3 |
|  | | T3 |  | 19,029 | 49.4 |  | 6,895 | 57.4 |  | 4,239 | 53.5 |
|  | | T4 |  | 6,068 | 15.7 |  | 3,655 | 30.4 |  | 3,208 | 40.5 |
|  | | TX |  | 57 | 0.1 |  | 14 | 0.1 |  | 14 | 0.2 |
| cN | | N0 |  | 24,661 | 64.0 |  | 6,488 | 54.0 |  | 4,077 | 51.4 |
|  | | N1 |  | 9,807 | 25.4 |  | 3,597 | 30.0 |  | 2,353 | 29.7 |
|  | | N2 |  | 4,019 | 10.4 |  | 1,889 | 15.7 |  | 1,440 | 18.2 |
|  | | NX |  | 51 | 0.1 |  | 36 | 0.3 |  | 58 | 0.7 |
| Preoperative treatment | | |  | 2,860 | 7.4 |  | 1,334 | 11.1 |  | 925 | 11.7 |
| Preoperative comorbidity | | | |  |  |  |  |  |  |  |  |
|  | Diabetes mellitus | |  | 7,665 | 19.9 |  | 2,651 | 22.1 |  | 1,677 | 21.2 |
|  | Hypertension | |  | 16,787 | 43.6 |  | 5,396 | 44.9 |  | 3,445 | 43.5 |
|  | COPD | |  | 1,113 | 2.9 |  | 440 | 3.7 |  | 298 | 3.8 |
|  | Cardiac disease | |  | 1,815 | 4.7 |  | 828 | 6.9 |  | 536 | 6.8 |
|  | Cerebrovascular disease | | | 1,432 | 3.7 |  | 761 | 6.3 |  | 490 | 6.2 |
|  | Kidney dysfunction | |  | 185 | 0.5 |  | 178 | 1.5 |  | 118 | 1.5 |
| GPS, Glasgow prognostic score; ASA-PS, American Society of Anesthesiologists - Physical Status; cT, preoperative diagnosis of tumor invasion depth; cN, preoperative diagnosis of lymph node metastasis; COPD, chronic obstructive pulmonary disease. | | | | | | | | | | | |
