# Supplementary material for: Significance of the Glasgow prognostic score for short‐term surgical outcomes: A nationwide survey using the Japanese National Clinical Database
Source: Ann Gastroenterol Surg. 2021 Mar 21;5(5):659–68. doi: 10.1002/ags3.12456 (PMC8452482; doi:10.1002/ags3.12456)
Supplement: Supplementary file 6 — Table S6 [file AGS3-5-659-s014.docx]

| **Table S6.** Background Parameters of Patients with Low Anterior Resection | | | | | | | | | | | |
| --- | --- | --- | --- | --- | --- | --- | --- | --- | --- | --- | --- |
|  | |  |  | **GPS** | | | | | | | |
|  | |  |  | **0 (n=51,831)** | |  | **1 (n=8,088)** | |  | **2 (n=2,774)** | |
| **Characteristics** | | |  | **n** | **%** |  | **n** | **%** |  | **n** | **%** |
| Age (years) | | <60 |  | 12,516 | 24.1 |  | 1,013 | 12.5 |  | 295 | 10.6 |
|  | | <70 |  | 17,976 | 34.7 |  | 2,251 | 27.8 |  | 721 | 26.0 |
|  | | <80 |  | 15,747 | 30.4 |  | 2,885 | 35.7 |  | 1,032 | 37.2 |
|  | | 80≤ |  | 5,592 | 10.8 |  | 1,939 | 24.0 |  | 726 | 26.2 |
| Sex | | Male |  | 33,774 | 65.2 |  | 5,334 | 65.9 |  | 1,846 | 66.5 |
|  | | Female |  | 18,057 | 34.8 |  | 2,754 | 34.1 |  | 928 | 33.5 |
| ASA-PS | | 1 |  | 14,882 | 28.7 |  | 1,303 | 16.1 |  | 375 | 13.5 |
|  | | 2 |  | 32,895 | 63.5 |  | 5,296 | 65.5 |  | 1,805 | 65.1 |
|  | | 3 |  | 3,983 | 7.7 |  | 1,457 | 18.0 |  | 580 | 20.9 |
|  | | 4 |  | 53 | 0.1 |  | 31 | 0.4 |  | 12 | 0.4 |
|  | | 5 |  | 17 | 0.0 |  | 1 | 0.0 |  | 2 | 0.1 |
| cT | | T0 |  | 296 | 0.6 |  | 37 | 0.5 |  | 9 | 0.3 |
|  | | Tis |  | 996 | 1.9 |  | 111 | 1.4 |  | 26 | 0.9 |
|  | | T1 |  | 9,725 | 18.8 |  | 652 | 8.1 |  | 121 | 4.4 |
|  | | T2 |  | 10,992 | 21.2 |  | 1,002 | 12.4 |  | 249 | 9.0 |
|  | | T3 |  | 24,015 | 46.3 |  | 4,528 | 56.0 |  | 1,488 | 53.6 |
|  | | T4 |  | 5,649 | 10.9 |  | 1,743 | 21.6 |  | 869 | 31.3 |
|  | | TX |  | 158 | 0.3 |  | 15 | 0.2 |  | 12 | 0.4 |
| cN | | N0 |  | 32,881 | 63.4 |  | 4,492 | 55.5 |  | 1,541 | 55.6 |
|  | | N1 |  | 13,172 | 25.4 |  | 2,371 | 29.3 |  | 808 | 29.1 |
|  | | N2 |  | 5,668 | 10.9 |  | 1,204 | 14.9 |  | 400 | 14.4 |
|  | | NX |  | 110 | 0.2 |  | 21 | 0.3 |  | 25 | 0.9 |
| Preoperative treatment | | |  | 7,460 | 14.4 |  | 1,426 | 17.6 |  | 511 | 18.4 |
| Preoperative comorbidity | | | |  |  |  |  |  |  |  |  |
|  | Diabetes mellitus | |  | 9,170 | 17.7 |  | 1,888 | 23.3 |  | 628 | 22.6 |
|  | Hypertension | |  | 18,895 | 36.5 |  | 3,425 | 42.3 |  | 1,118 | 40.3 |
|  | COPD | |  | 1,767 | 3.4 |  | 400 | 4.9 |  | 113 | 4.1 |
|  | Cardiac disease | |  | 1,904 | 3.7 |  | 492 | 6.1 |  | 209 | 7.5 |
|  | Cerebrovascular disease | | | 1,538 | 3.0 |  | 454 | 5.6 |  | 180 | 6.5 |
|  | Kidney dysfunction | |  | 174 | 0.3 |  | 141 | 1.7 |  | 47 | 1.7 |
| GPS, Glasgow prognostic score; ASA-PS, American Society of Anesthesiologists - Physical Status; cT, preoperative diagnosis of tumor invasion depth; cN, preoperative diagnosis of lymph node metastasis; COPD, chronic obstructive pulmonary disease. | | | | | | | | | | | |
