# Supplementary material for: Significance of the Glasgow prognostic score for short‐term surgical outcomes: A nationwide survey using the Japanese National Clinical Database
Source: Ann Gastroenterol Surg. 2021 Mar 21;5(5):659–68. doi: 10.1002/ags3.12456 (PMC8452482; doi:10.1002/ags3.12456)
Supplement: Supplementary file 7 — Table S7 [file AGS3-5-659-s011.docx]

| **Table S7.** Background Parameters of Patients with Pancreaticoduodenectomy | | | | | | | | | | | |
| --- | --- | --- | --- | --- | --- | --- | --- | --- | --- | --- | --- |
|  | |  |  | **GPS** | | | | | | | |
|  | |  |  | **0 (n=13,901)** | |  | **1 (n=4,450)** | |  | **2 (n=1,617)** | |
| **Characteristics** | | |  | **n** | **%** |  | **n** | **%** |  | **n** | **%** |
| Age (years) | | <60 |  | 2,196 | 15.8 |  | 429 | 9.6 |  | 122 | 7.5 |
|  | | <70 |  | 4,499 | 32.4 |  | 1,292 | 29.0 |  | 416 | 25.7 |
|  | | <80 |  | 5,699 | 41.0 |  | 2,037 | 45.8 |  | 766 | 47.4 |
|  | | 80≤ |  | 1,507 | 10.8 |  | 692 | 15.6 |  | 313 | 19.4 |
| Sex | | Male |  | 7,706 | 55.4 |  | 2,588 | 58.2 |  | 920 | 56.9 |
|  | | Female |  | 6,195 | 44.6 |  | 1,862 | 41.8 |  | 697 | 43.1 |
| ASA-PS | | 1 |  | 2,359 | 17.0 |  | 453 | 10.2 |  | 131 | 8.1 |
|  | | 2 |  | 9,987 | 71.8 |  | 3,266 | 73.4 |  | 1,167 | 72.2 |
|  | | 3 |  | 1,535 | 11.0 |  | 723 | 16.2 |  | 312 | 19.3 |
|  | | 4 |  | 14 | 0.1 |  | 6 | 0.1 |  | 4 | 0.2 |
|  | | 5 |  | 6 | 0.0 |  | 2 | 0.0 |  | 3 | 0.2 |
| cT | | T0 |  | 71 | 0.5 |  | 12 | 0.3 |  | 4 | 0.2 |
|  | | Tis |  | 1,004 | 7.2 |  | 95 | 2.1 |  | 25 | 1.5 |
|  | | T1 |  | 1,916 | 13.8 |  | 319 | 7.2 |  | 86 | 5.3 |
|  | | T2 |  | 2,725 | 19.6 |  | 774 | 17.4 |  | 250 | 15.5 |
|  | | T3 |  | 7,351 | 52.9 |  | 2,904 | 65.3 |  | 1,122 | 69.4 |
|  | | T4 |  | 802 | 5.8 |  | 334 | 7.5 |  | 127 | 7.9 |
|  | | TX |  | 32 | 0.2 |  | 12 | 0.3 |  | 3 | 0.2 |
| cN | | N0 |  | 7,848 | 56.5 |  | 2,025 | 45.5 |  | 665 | 41.1 |
|  | | N1 |  | 5,708 | 41.1 |  | 2,283 | 51.3 |  | 892 | 55.2 |
|  | | N2 |  | 322 | 2.3 |  | 134 | 3.0 |  | 54 | 3.3 |
|  | | NX |  | 23 | 0.2 |  | 8 | 0.2 |  | 6 | 0.4 |
| Preoperative treatment | | |  | 3,152 | 22.7 |  | 1,425 | 32.0 |  | 529 | 32.7 |
| Preoperative comorbidity | | | |  |  |  |  |  |  |  |  |
|  | Diabetes mellitus | |  | 5,012 | 36.1 |  | 1,724 | 38.7 |  | 643 | 39.8 |
|  | Hypertension | |  | 5,355 | 38.5 |  | 1,874 | 42.1 |  | 725 | 44.8 |
|  | COPD | |  | 474 | 3.4 |  | 162 | 3.6 |  | 76 | 4.7 |
|  | Cardiac disease | |  | 549 | 3.9 |  | 210 | 4.7 |  | 117 | 7.2 |
|  | Cerebrovascular disease | | | 401 | 2.9 |  | 177 | 4.0 |  | 71 | 4.4 |
|  | Kidney dysfunction | |  | 46 | 0.33 |  | 24 | 0.5 |  | 22 | 1.4 |
| GPS, Glasgow prognostic score; ASA-PS, American Society of Anesthesiologists - Physical Status; cT, preoperative diagnosis of tumor invasion depth; cN, preoperative diagnosis of lymph node metastasis; COPD, chronic obstructive pulmonary disease. | | | | | | | | | | | |
