# Supplementary material for: Significance of the Glasgow prognostic score for short‐term surgical outcomes: A nationwide survey using the Japanese National Clinical Database
Source: Ann Gastroenterol Surg. 2021 Mar 21;5(5):659–68. doi: 10.1002/ags3.12456 (PMC8452482; doi:10.1002/ags3.12456)
Supplement: Supplementary file 8 — Table S8 [file AGS3-5-659-s001.docx]

| **Table S8.** Preoperative Treatment for Each Procedure | | | | |
| --- | --- | --- | --- | --- |
|  | **Preoperative treatment** | **Number** | **%** |  |
| Eso | Chemoradiotherapy | 1262 | 6.1% |  |
|  | Chemotherapy | 9175 | 44.7% |  |
|  | Radiotherapy | 82 | 0.4% |  |
| TG | Chemoradiotherapy | 42 | 0.1% |  |
|  | Chemotherapy | 3,053 | 7.4% |  |
|  | Radiotherapy | 19 | 0.05% |  |
| DG | Chemoradiotherapy | 64 | 0.1% |  |
|  | Chemotherapy | 2,482 | 2.3% |  |
|  | Radiotherapy | 34 | 0.03% |  |
| RHC | Chemoradiotherapy | 17 | 0.03% |  |
|  | Chemotherapy | 480 | 0.8% |  |
|  | Radiotherapy | 57 | 0.1% |  |
| LAR | Chemoradiotherapy | 1,903 | 3.0% |  |
|  | Chemotherapy | 2,492 | 4.0% |  |
|  | Radiotherapy | 243 | 0.4% |  |
| PD | Chemoradiotherapy | 841 | 4.2% |  |
|  | Chemotherapy | 1,881 | 9.4% |  |
|  | Radiotherapy | 38 | 0.2% |  |
| Eso, esophagectomy; TG, total gastrectomy; DG, distal gastrectomy; RHC, right hemicolectomy; LAR, low anterior resection; PD, pancreaticoduodenectomy. | | | | |
