# Supplementary material for: Significance of the Glasgow prognostic score for short‐term surgical outcomes: A nationwide survey using the Japanese National Clinical Database
Source: Ann Gastroenterol Surg. 2021 Mar 21;5(5):659–68. doi: 10.1002/ags3.12456 (PMC8452482; doi:10.1002/ags3.12456)
Supplement: Supplementary file 9 — Table S9 [file AGS3-5-659-s005.docx]

| **Table S9.** Estimates from Multivariable Logistic Regression for Operative Morbidity and Mortality after Esophagectomy | | | | | | | | | | |
| --- | --- | --- | --- | --- | --- | --- | --- | --- | --- | --- |
|  | | |  | **Complication CD3 and above** | | |  | **Operative Death** | | |
|  | | |  | **OR** | **95% CI** | ***P*-value** |  | **OR** | **95% CI** | ***P*-value** |
| GPS | | 1 vs. 0 |  | 1.15 | (1.04-1.26) | 0.005 |  | 1.57 | (1.20-2.04) | <0.001 |
|  | | 2 vs. 0 |  | 1.42 | (1.22-1.66) | <.0001 |  | 2.62 | (1.88-3.65) | <0.001 |
| Age | | <70 vs. <60 |  | 0.97 | (0.88-1.08) | 0.61 |  | 2.49 | (1.47-4.23) | <0.001 |
|  | | <80 vs. <60 |  | 1.03 | (0.93-1.14) | 0.63 |  | 4.06 | (2.41-6.83) | <0.001 |
|  | | 80 - vs. <60 |  | 1.09 | (0.92-1.29) | 0.32 |  | 6.65 | (3.70-11.95) | <0.001 |
| Sex | | Male vs. female |  | 1.16 | (1.06-1.27) | 0.002 |  | 1.90 | (1.33-2.73) | <0.001 |
| ASA-PS | | 2 vs. 1 |  | 1.41 | (1.27-1.55) | <.0001 |  | 1.62 | (1.11-2.38) | 0.01 |
|  | | 3 vs. 1 |  | 1.74 | (1.51-2.02) | <.0001 |  | 2.91 | (1.86-4.56) | <0.001 |
|  | | 4 vs. 1 |  | 1.74 | (0.66-4.62) | 0.27 |  | 4.52 | (0.90-22.73) | 0.07 |
|  | | 5 vs. 1 |  | 0.84 | (0.19-3.74) | 0.82 |  | - | - | - |
| cT | | T0 vs. T1 |  | 0.98 | (0.75-1.27) | 0.87 |  | 1.46 | (0.62-3.41) | 0.39 |
|  | | T2 vs. T1 |  | 0.95 | (0.85-1.06) | 0.37 |  | 1.14 | (0.78-1.66) | 0.49 |
|  | | T3 vs. T1 |  | 1.04 | (0.95-1.14) | 0.35 |  | 1.38 | (1.02-1.85) | 0.04 |
|  | | T4 vs. T1 |  | 1.33 | (1.14-1.54) | <0.001 |  | 2.54 | (1.71-3.79) | <0.001 |
|  | | TX vs. T1 |  | 2.39 | (1.39-4.10) | 0.00 |  | 3.04 | (0.71-13.00) | 0.13 |
|  | | Tis vs. T1 |  | 1.01 | (0.62-1.66) | 0.96 |  | 0.85 | (0.19-3.90) | 0.83 |
| cN | | N1 vs. N0 |  | 1.05 | (0.97-1.15) | 0.25 |  | 1.13 | (0.86-1.49) | 0.38 |
|  | | N2 vs. N0 |  | 1.04 | (0.94-1.15) | 0.44 |  | 1.25 | (0.92-1.70) | 0.15 |
|  | | N3 vs. N0 |  | 0.91 | (0.78-1.06) | 0.21 |  | 1.45 | (0.96-2.17) | 0.08 |
|  | | NX vs. N0 |  | 1.54 | (0.92-2.57) | 0.10 |  | 5.38 | (2.31-12.49) | <0.001 |
| Preoperative treatment | | |  | 1.03 | (0.95-1.11) | 0.50 |  | 0.89 | (0.71-1.12) | 0.31 |
| Preoperative comorbidity | | |  |  |  |  |  |  |  |  |
|  | Diabetes mellitus | |  | 1.11 | (1.01-1.22) | 0.04 |  | 1.26 | (0.97-1.63) | 0.08 |
|  | Hypertension | |  | 1.04 | (0.97-1.12) | 0.32 |  | 0.97 | (0.78-1.21) | 0.80 |
|  | Cardiac disease | |  | 1.10 | (0.92-1.32) | 0.30 |  | 1.32 | (0.87-2.01) | 0.19 |
|  | Kidney dysfunction | |  | 0.92 | (0.53-1.61) | 0.77 |  | 3.25 | (1.45-7.26) | 0.004 |
|  | Cerebrovascular disease | |  | 1.36 | (1.13-1.64) | 0.001 |  | 1.45 | (0.92-2.28) | 0.11 |
|  | COPD | |  | 1.51 | (1.35-1.69) | <.0001 |  | 1.86 | (1.40-2.49) | <0.001 |
| CD, Clavien-Dindo classification; OR, odds ratio; CI, confidence interval; GPS, Glasgow prognostic score; ASA-PS, American Society of Anesthesiologists - Physical Status; cT, preoperative diagnosis of tumor invasion depth; cN, preoperative diagnosis of lymph node metastasis; COPD, chronic obstructive pulmonary disease. | | | | | | | | | | |
