# Supplementary material for: Significance of the Glasgow prognostic score for short‐term surgical outcomes: A nationwide survey using the Japanese National Clinical Database
Source: Ann Gastroenterol Surg. 2021 Mar 21;5(5):659–68. doi: 10.1002/ags3.12456 (PMC8452482; doi:10.1002/ags3.12456)
Supplement: Supplementary file 10 — Table S10 [file AGS3-5-659-s010.docx]

| **Table S10.** Estimates from Multivariable Logistic Regression for Operative Morbidity and Mortality after Total Gastrectomy | | | | | | | | | | |
| --- | --- | --- | --- | --- | --- | --- | --- | --- | --- | --- |
|  | | |  | **Complication CD3 and above** | | |  | **Operative Death** | | |
|  | | |  | **OR** | **95% CI** | ***P*-value** |  | **OR** | **95% CI** | ***P*-value** |
| GPS | | 1 vs. 0 |  | 1.11 | (1.02-1.22) | 0.02 |  | 2.11 | (1.71-2.59) | <.0001 |
|  | | 2 vs. 0 |  | 1.31 | (1.16-1.48) | <.0001 |  | 3.07 | (2.40-3.92) | <.0001 |
| Age | | <70 vs. <60 |  | 0.96 | (0.85-1.09) | 0.56 |  | 2.12 | (1.17-3.82) | 0.01 |
|  | | <80 vs. <60 |  | 0.96 | (0.85-1.09) | 0.54 |  | 2.84 | (1.60-5.04) | 0.0004 |
|  | | 80 - vs. <60 |  | 1.00 | (0.87-1.15) | 0.99 |  | 6.80 | (3.83-12.05) | <.0001 |
| Sex | | Male vs. female |  | 1.66 | (1.51-1.81) | <.0001 |  | 1.59 | (1.25-2.01) | 0.0001 |
| ASA-PS | | 2 vs. 1 |  | 1.42 | (1.28-1.57) | <.0001 |  | 1.28 | (0.92-1.76) | 0.14 |
|  | | 3 vs. 1 |  | 1.54 | (1.34-1.77) | <.0001 |  | 2.15 | (1.50-3.09) | <.0001 |
|  | | 4 vs. 1 |  | 1.94 | (1.07-3.50) | 0.03 |  | 3.16 | (1.26-7.94) | 0.01 |
|  | | 5 vs. 1 |  | - | - | - |  | - | - | - |
| cT | | T0 vs. T1 |  | 0.74 | (0.32-1.69) | 0.47 |  | 2.84 | (0.67-12.02) | 0.16 |
|  | | T2 vs. T1 |  | 1.08 | (0.95-1.21) | 0.24 |  | 1.07 | (0.76-1.49) | 0.70 |
|  | | T3 vs. T1 |  | 1.16 | (1.04-1.29) | 0.01 |  | 1.06 | (0.79-1.42) | 0.72 |
|  | | T4 vs. T1 |  | 1.42 | (1.27-1.59) | <.0001 |  | 1.27 | (0.94-1.72) | 0.12 |
|  | | TX vs. T1 |  | 0.62 | (0.22-1.73) | 0.36 |  | 5.56 | (1.99-15.5) | 0.001 |
|  | | Tis vs. T1 |  | 1.39 | (0.76-2.53) | 0.29 |  | 1.15 | (0.16-8.48) | 0.89 |
| cN | | N1 vs. N0 |  | 1.13 | (1.02-1.25) | 0.02 |  | 1.21 | (0.93-1.58) | 0.16 |
|  | | N2 vs. N0 |  | 1.06 | (0.95-1.18) | 0.31 |  | 1.26 | (0.96-1.67) | 0.10 |
|  | | N3 vs. N0 |  | 0.95 | (0.85-1.07) | 0.41 |  | 1.44 | (1.09-1.90) | 0.01 |
|  | | NX vs. N0 |  | 1.45 | (0.79-2.64) | 0.23 |  | 5.09 | (2.40-10.79) | <.0001 |
| Preoperative treatment | | |  | 1.07 | (0.97-1.18) | 0.18 |  | 0.81 | (0.61-1.08) | 0.15 |
| Preoperative comorbidity | | |  |  |  |  |  |  |  |  |
|  | Diabetes mellitus | |  | 1.02 | (0.94-1.11) | 0.65 |  | 1.05 | (0.86-1.29) | 0.64 |
|  | Hypertension | |  | 1.20 | (1.11-1.29) | <.0001 |  | 1.03 | (0.86-1.23) | 0.76 |
|  | Cardiac disease | |  | 1.50 | (1.32-1.70) | <.0001 |  | 1.79 | (1.38-2.31) | <.0001 |
|  | Kidney dysfunction | |  | 1.88 | (1.38-2.56) | <.0001 |  | 3.55 | (2.24-5.64) | <.0001 |
|  | Cerebrovascular disease | |  | 1.26 | (1.09-1.46) | 0.002 |  | 1.30 | (0.95-1.77) | 0.10 |
|  | COPD | |  | 1.25 | (1.09-1.42) | 0.001 |  | 1.73 | (1.31-2.28) | <.0001 |
| CD, Clavien-Dindo classification; OR, odds ratio; CI, confidence interval; GPS, Glasgow prognostic score; ASA-PS, American Society of Anesthesiologists - Physical Status; cT, preoperative diagnosis of tumor invasion depth; cN, preoperative diagnosis of lymph node metastasis; COPD, chronic obstructive pulmonary disease. | | | | | | | | | | |
