# Supplementary material for: Significance of the Glasgow prognostic score for short‐term surgical outcomes: A nationwide survey using the Japanese National Clinical Database
Source: Ann Gastroenterol Surg. 2021 Mar 21;5(5):659–68. doi: 10.1002/ags3.12456 (PMC8452482; doi:10.1002/ags3.12456)
Supplement: Supplementary file 11 — Table S11 [file AGS3-5-659-s015.docx]

| **Table S11.** Estimates from Multivariable Logistic Regression for Operative Morbidity and Mortality after Distal Gastrectomy | | | | | | | | | | |
| --- | --- | --- | --- | --- | --- | --- | --- | --- | --- | --- |
|  | | |  | **Complication CD3 and above** | | |  | **Operative Death** | | |
|  | | |  | **OR** | **95% CI** | ***P*-value** |  | **OR** | **95% CI** | ***P*-value** |
| GPS | | 1 vs. 0 |  | 1.26 | (1.17-1.35) | <.0001 |  | 2.21 | (1.87-2.62) | <.0001 |
|  | | 2 vs. 0 |  | 1.43 | (1.29-1.60) | <.0001 |  | 3.83 | (3.14-4.67) | <.0001 |
| Age | | <70 vs. <60 |  | 1.08 | (0.98-1.20) | 0.13 |  | 2.02 | (1.17-3.52) | 0.01 |
|  | | <80 vs. <60 |  | 1.20 | (1.08-1.33) | 0.001 |  | 3.84 | (2.26-6.50) | <.0001 |
|  | | 80 - vs. <60 |  | 1.35 | (1.21-1.50) | <.0001 |  | 8.44 | (4.99-14.29) | <.0001 |
| Sex | | Male vs. female |  | 1.70 | (1.59-1.81) | <.0001 |  | 1.78 | (1.50-2.11) | <.0001 |
| ASA-PS | | 2 vs. 1 |  | 1.43 | (1.32-1.56) | <.0001 |  | 1.40 | (1.05-1.85) | 0.02 |
|  | | 3 vs. 1 |  | 1.86 | (1.67-2.06) | <.0001 |  | 2.53 | (1.87-3.43) | <.0001 |
|  | | 4 vs. 1 |  | 2.60 | (1.70-3.98) | <.0001 |  | 6.03 | (3.22-11.3) | <.0001 |
|  | | 5 vs. 1 |  | 1.47 | (0.35-6.18) | 0.60 |  | - | - | - |
| cT | | T0 vs. T1 |  | 1.00 | (0.59-1.69) | 1.00 |  | 1.71 | (0.42-6.97) | 0.45 |
|  | | T2 vs. T1 |  | 1.15 | (1.06-1.25) | 0.001 |  | 1.21 | (0.96-1.52) | 0.11 |
|  | | T3 vs. T1 |  | 1.21 | (1.11-1.32) | <.0001 |  | 1.32 | (1.06-1.64) | 0.01 |
|  | | T4 vs. T1 |  | 1.44 | (1.31-1.58) | <.0001 |  | 1.68 | (1.33-2.11) | <.0001 |
|  | | TX vs. T1 |  | 1.33 | (0.72-2.47) | 0.36 |  | 1.57 | (0.43-5.77) | 0.50 |
|  | | Tis vs. T1 |  | 1.29 | (0.88-1.89) | 0.20 |  | 4.80 | (2.50-9.19) | <.0001 |
| cN | | N1 vs. N0 |  | 1.03 | (0.95-1.12) | 0.44 |  | 1.04 | (0.84-1.28) | 0.75 |
|  | | N2 vs. N0 |  | 1.10 | (1.00-1.20) | 0.06 |  | 1.14 | (0.91-1.42) | 0.27 |
|  | | N3 vs. N0 |  | 1.06 | (0.96-1.18) | 0.27 |  | 1.26 | (0.99-1.60) | 0.06 |
|  | | NX vs. N0 |  | 1.02 | (0.64-1.64) | 0.92 |  | 2.51 | (1.30-4.83) | 0.01 |
| Preoperative treatment | | |  | 1.07 | (0.98-1.17) | 0.12 |  | 0.74 | (0.58-0.96) | 0.02 |
| Preoperative comorbidity | | |  |  |  |  |  |  |  |  |
|  | Diabetes mellitus | |  | 1.08 | (1.01-1.15) | 0.03 |  | 1.07 | (0.91-1.26) | 0.42 |
|  | Hypertension | |  | 1.11 | (1.05-1.18) | 0.0003 |  | 1.06 | (0.92-1.22) | 0.45 |
|  | Cardiac disease | |  | 1.36 | (1.23-1.49) | <.0001 |  | 1.89 | (1.57-2.28) | <.0001 |
|  | Kidney dysfunction | |  | 2.06 | (1.69-2.50) | <.0001 |  | 4.42 | (3.26-6.01) | <.0001 |
|  | Cerebrovascular disease | |  | 1.40 | (1.26-1.56) | <.0001 |  | 1.47 | (1.17-1.85) | 0.001 |
|  | COPD | |  | 1.35 | (1.22-1.49) | <.0001 |  | 1.15 | (0.90-1.48) | 0.27 |
| CD, Clavien-Dindo classification; OR, odds ratio; CI, confidence interval; GPS, Glasgow prognostic score; ASA-PS, American Society of Anesthesiologists - Physical Status; cT, preoperative diagnosis of tumor invasion depth; cN, preoperative diagnosis of lymph node metastasis; COPD, chronic obstructive pulmonary disease. | | | | | | | | | | |
