# Supplementary material for: Significance of the Glasgow prognostic score for short‐term surgical outcomes: A nationwide survey using the Japanese National Clinical Database
Source: Ann Gastroenterol Surg. 2021 Mar 21;5(5):659–68. doi: 10.1002/ags3.12456 (PMC8452482; doi:10.1002/ags3.12456)
Supplement: Supplementary file 12 — Table S12 [file AGS3-5-659-s006.docx]

| **Table S12.** Estimates from Multivariable Logistic Regression for Operative Morbidity and Mortality after Right Hemicolectomy | | | | | | | | | | |
| --- | --- | --- | --- | --- | --- | --- | --- | --- | --- | --- |
|  | | |  | **Complication CD3 and above** | | |  | **Operative Death** | | |
|  | | |  | **OR** | **95% CI** | ***P*-value** |  | **OR** | **95% CI** | ***P*-value** |
| GPS | | 1 vs. 0 |  | 1.27 | (1.15-1.41) | <.0001 |  | 2.59 | (1.94-3.44) | <.0001 |
|  | | 2 vs. 0 |  | 1.68 | (1.50-1.88) | <.0001 |  | 5.63 | (4.28-7.42) | <.0001 |
| Age | | <70 vs. <60 |  | 0.90 | (0.75-1.07) | 0.23 |  | 0.66 | (0.33-1.34) | 0.25 |
|  | | <80 vs. <60 |  | 1.02 | (0.87-1.21) | 0.79 |  | 1.50 | (0.81-2.75) | 0.19 |
|  | | 80 - vs. <60 |  | 1.17 | (0.99-1.39) | 0.07 |  | 3.42 | (1.88-6.2) | <.0001 |
| Sex | | Male vs. female |  | 1.90 | (1.74-2.08) | <.0001 |  | 1.64 | (1.33-2.03) | <.0001 |
| ASA-PS | | 2 vs. 1 |  | 1.64 | (1.43-1.88) | <.0001 |  | 1.76 | (1.09-2.84) | 0.02 |
|  | | 3 vs. 1 |  | 1.88 | (1.59-2.23) | <.0001 |  | 3.67 | (2.23-6.05) | <.0001 |
|  | | 4 vs. 1 |  | 4.77 | (2.98-7.65) | <.0001 |  | 15.19 | (7.27-31.7) | <.0001 |
|  | | 5 vs. 1 |  | 3.64 | (1.09-12.15) | 0.04 |  | - | - | - |
| cT | | T0 vs. T1 |  | 1.88 | (0.93-3.78) | 0.08 |  | 1.21 | (0.15-9.66) | 0.86 |
|  | | T2 vs. T1 |  | 1.08 | (0.90-1.30) | 0.41 |  | 0.67 | (0.39-1.15) | 0.15 |
|  | | T3 vs. T1 |  | 1.10 | (0.95-1.28) | 0.22 |  | 0.76 | (0.50-1.15) | 0.20 |
|  | | T4 vs. T1 |  | 1.38 | (1.16-1.63) | 0.0002 |  | 0.76 | (0.48-1.20) | 0.24 |
|  | | TX vs. T1 |  | 0.85 | (0.29-2.47) | 0.76 |  | 0.43 | (0.05-3.61) | 0.44 |
|  | | Tis vs. T1 |  | 1.14 | (0.86-1.51) | 0.38 |  | 0.75 | (0.31-1.83) | 0.52 |
| cN | | N1 vs. N0 |  | 0.97 | (0.88-1.07) | 0.50 |  | 1.04 | (0.82-1.34) | 0.73 |
|  | | N2 vs. N0 |  | 0.88 | (0.77-1.01) | 0.06 |  | 1.47 | (1.09-1.97) | 0.01 |
|  | | NX vs. N0 |  | 2.16 | (1.23-3.77) | 0.01 |  | 8.92 | (4.70-16.9) | <.0001 |
| Preoperative treatment | | |  | 1.25 | (1.10-1.42) | 0.001 |  | 1.25 | (1.10-1.42) | 0.001 |
| Preoperative comorbidity | | |  |  |  |  |  |  |  |  |
|  | Diabetes mellitus | |  | 0.98 | (0.89-1.08) | 0.66 |  | 0.98 | (0.89-1.08) | 0.66 |
|  | Hypertension | |  | 1.06 | (0.98-1.16) | 0.17 |  | 1.06 | (0.98-1.16) | 0.17 |
|  | Cardiac disease | |  | 1.12 | (0.96-1.32) | 0.15 |  | 1.12 | (0.96-1.32) | 0.15 |
|  | Kidney dysfunction | |  | 1.56 | (1.12-2.17) | 0.01 |  | 1.56 | (1.12-2.17) | 0.01 |
|  | Cerebrovascular disease | |  | 1.25 | (1.07-1.48) | 0.01 |  | 1.25 | (1.07-1.48) | 0.01 |
|  | COPD | |  | 1.44 | (1.20-1.72) | <.0001 |  | 1.44 | (1.20-1.72) | <.0001 |
| CD, Clavien-Dindo classification; OR, odds ratio; CI, confidence interval; GPS, Glasgow prognostic score; ASA-PS, American Society of Anesthesiologists - Physical Status; cT, preoperative diagnosis of tumor invasion depth; cN, preoperative diagnosis of lymph node metastasis; COPD, chronic obstructive pulmonary disease. | | | | | | | | | | |
