# Supplementary material for: Significance of the Glasgow prognostic score for short‐term surgical outcomes: A nationwide survey using the Japanese National Clinical Database
Source: Ann Gastroenterol Surg. 2021 Mar 21;5(5):659–68. doi: 10.1002/ags3.12456 (PMC8452482; doi:10.1002/ags3.12456)
Supplement: Supplementary file 13 — Table S13 [file AGS3-5-659-s004.docx]

| **Table S13.** Estimates from Multivariable Logistic Regression for Operative Morbidity and Mortality after Low Anterior Resection | | | | | | | | | | |
| --- | --- | --- | --- | --- | --- | --- | --- | --- | --- | --- |
|  | | |  | **Complication CD3 and above** | | |  | **Operative Death** | | |
|  | | |  | **OR** | **95% CI** | ***P*-value** |  | **OR** | **95% CI** | ***P*-value** |
| GPS | | 1 vs. 0 |  | 1.32 | (1.22-1.42) | <.0001 |  | 2.11 | (1.58-2.80) | <.0001 |
|  | | 2 vs. 0 |  | 1.58 | (1.42-1.77) | <.0001 |  | 5.59 | (4.15-7.54) | <.0001 |
| Age | | <70 vs. <60 |  | 0.86 | (0.80-0.92) | <.0001 |  | 1.42 | (0.84-2.42) | 0.19 |
|  | | <80 vs. <60 |  | 0.79 | (0.73-0.85) | <.0001 |  | 2.69 | (1.63-4.44) | 0.0001 |
|  | | 80 - vs. <60 |  | 0.75 | (0.68-0.82) | <.0001 |  | 5.74 | (3.45-9.56) | <.0001 |
| Sex | | Male vs. female |  | 1.96 | (1.84-2.09) | <.0001 |  | 1.53 | (1.17-1.99) | 0.002 |
| ASA-PS | | 2 vs. 1 |  | 1.29 | (1.20-1.38) | <.0001 |  | 1.34 | (0.91-1.98) | 0.14 |
|  | | 3 vs. 1 |  | 1.47 | (1.32-1.63) | <.0001 |  | 2.78 | (1.80-4.32) | <.0001 |
|  | | 4 vs. 1 |  | 2.03 | (1.19-3.48) | 0.01 |  | 5.80 | (1.89-17.83) | 0.002 |
|  | | 5 vs. 1 |  | 1.02 | (0.24-4.44) | 0.98 |  | - | - | - |
| cT | | T0 vs. T1 |  | 0.92 | (0.62-1.38) | 0.68 |  | 1.18 | (0.16-8.80) | 0.87 |
|  | | T2 vs. T1 |  | 1.13 | (1.03-1.24) | 0.01 |  | 1.18 | (0.74-1.90) | 0.48 |
|  | | T3 vs. T1 |  | 1.32 | (1.22-1.44) | <.0001 |  | 1.31 | (0.86-1.99) | 0.21 |
|  | | T4 vs. T1 |  | 1.31 | (1.18-1.46) | <.0001 |  | 1.23 | (0.75-2.01) | 0.42 |
|  | | TX vs. T1 |  | 1.03 | (0.59-1.78) | 0.93 |  | - | - | - |
|  | | Tis vs. T1 |  | 0.81 | (0.64-1.04) | 0.10 |  | 0.49 | (0.12-2.07) | 0.33 |
| cN | | N1 vs. N0 |  | 1.05 | (0.99-1.12) | 0.14 |  | 1.16 | (0.89-1.52) | 0.27 |
|  | | N2 vs. N0 |  | 1.23 | (1.13-1.33) | <.0001 |  | 1.22 | (0.85-1.75) | 0.28 |
|  | | NX vs. N0 |  | 1.11 | (0.64-1.92) | 0.72 |  | 3.64 | (1.07-12.4) | 0.04 |
| Preoperative treatment | | |  | 1.07 | (1.00-1.15) | 0.05 |  | 0.94 | (0.67-1.31) | 0.70 |
| Preoperative comorbidity | | |  |  |  |  |  |  |  |  |
|  | Diabetes mellitus | |  | 0.94 | (0.88-1.01) | 0.09 |  | 0.88 | (0.67-1.17) | 0.37 |
|  | Hypertension | |  | 1.04 | (0.98-1.10) | 0.17 |  | 0.94 | (0.74-1.19) | 0.59 |
|  | Cardiac disease | |  | 1.02 | (0.90-1.16) | 0.72 |  | 1.37 | (0.95-1.99) | 0.10 |
|  | Kidney dysfunction | |  | 1.25 | (0.94-1.68) | 0.13 |  | 1.80 | (0.86-3.79) | 0.12 |
|  | Cerebrovascular disease | |  | 1.33 | (1.18-1.51) | <.0001 |  | 1.39 | (0.92-2.10) | 0.12 |
|  | COPD | |  | 1.09 | (0.96-1.24) | 0.18 |  | 1.95 | (1.32-2.87) | 0.001 |
| CD, Clavien-Dindo classification; OR, odds ratio; CI, confidence interval; GPS, Glasgow prognostic score; ASA-PS, American Society of Anesthesiologists - Physical Status; cT, preoperative diagnosis of tumor invasion depth; cN, preoperative diagnosis of lymph node metastasis; COPD, chronic obstructive pulmonary disease. | | | | | | | | | | |
