# Supplementary material for: Significance of the Glasgow prognostic score for short‐term surgical outcomes: A nationwide survey using the Japanese National Clinical Database
Source: Ann Gastroenterol Surg. 2021 Mar 21;5(5):659–68. doi: 10.1002/ags3.12456 (PMC8452482; doi:10.1002/ags3.12456)
Supplement: Supplementary file 14 — Table S14 [file AGS3-5-659-s012.docx]

| **Table S14.** Estimates from Multivariable Logistic Regression for Operative Morbidity and Mortality after Pancreaticoduodenectomy | | | | | | | | | | | | | |
| --- | --- | --- | --- | --- | --- | --- | --- | --- | --- | --- | --- | --- | --- |
|  | | |  | **Complication CD3 and above** | | | |  | | **Operative Death** | | | |
|  | | |  | **OR** | **95% CI** | ***P*-value** |  | | **OR** | | **95% CI** | ***P*-value** |  |
| GPS | | 1 vs. 0 |  | 1.06 | (0.96-1.16) | 0.26 |  | | 1.68 | | (1.30-2.16) | <.0001 |  |
|  | | 2 vs. 0 |  | 1.15 | (0.99-1.32) | 0.06 |  | | 2.06 | | (1.49-2.85) | <.0001 |  |
| Age | | <70 vs. <60 |  | 0.99 | (0.87-1.13) | 0.90 |  | | 1.91 | | (1.11-3.30) | 0.02 |  |
|  | | <80 vs. <60 |  | 1.09 | (0.96-1.24) | 0.18 |  | | 2.74 | | (1.62-4.64) | 0.0002 |  |
|  | | 80 - vs. <60 |  | 1.09 | (0.93-1.28) | 0.29 |  | | 4.18 | | (2.38-7.32) | <.0001 |  |
| Sex | | Male vs. female |  | 1.54 | (1.42-1.68) | <.0001 |  | | 1.45 | | (1.14-1.83) | 0.002 |  |
| ASA-PS | | 2 vs. 1 |  | 1.24 | (1.10-1.41) | 0.001 |  | | 0.97 | | (0.66-1.44) | 0.89 |  |
|  | | 3 vs. 1 |  | 1.38 | (1.17-1.62) | 0.0001 |  | | 1.49 | | (0.95-2.34) | 0.08 |  |
|  | | 4 vs. 1 |  | 1.94 | (0.76-4.98) | 0.17 |  | | 1.86 | | (0.24-14.66) | 0.56 |  |
|  | | 5 vs. 1 |  | - | - | - |  | | - | | - | - |  |
| cT | | T0 vs. T1 |  | 1.53 | (0.92-2.54) | 0.10 |  | | 1.16 | | (0.16-8.74) | 0.88 |  |
|  | | T2 vs. T1 |  | 0.89 | (0.77-1.03) | 0.11 |  | | 1.17 | | (0.73-1.86) | 0.51 |  |
|  | | T3 vs. T1 |  | 0.83 | (0.73-0.94) | 0.004 |  | | 1.00 | | (0.65-1.53) | 0.99 |  |
|  | | T4 vs. T1 |  | 1.02 | (0.85-1.24) | 0.81 |  | | 2.99 | | (1.84-4.84) | <.0001 |  |
|  | | TX vs. T1 |  | 1.20 | (0.57-2.53) | 0.63 |  | | - | | - | - |  |
|  | | Tis vs. T1 |  | 1.16 | (0.96-1.40) | 0.12 |  | | 0.65 | | (0.29-1.44) | 0.29 |  |
| cN | | N1 vs. N0 |  | 0.89 | (0.82-0.97) | 0.01 |  | | 1.34 | | (1.06-1.70) | 0.01 |  |
|  | | N2 vs. N0 |  | 1.08 | (0.84-1.37) | 0.55 |  | | 0.90 | | (0.42-1.96) | 0.80 |  |
|  | | NX vs. N0 |  | 1.49 | (0.67-3.30) | 0.33 |  | | 3.95 | | (0.86-18.1) | 0.08 |  |
| Preoperative treatment | | |  | 1.06 | (0.97-1.17) | 0.18 |  | | 1.16 | | (0.91-1.49) | 0.24 |  |
| Preoperative comorbidity | | |  |  |  |  |  | |  | |  |  |  |
|  | Diabetes mellitus | |  | 0.82 | (0.75-0.89) | <.0001 |  | | 1.10 | | (0.87-1.38) | 0.44 |  |
|  | Hypertension | |  | 1.13 | (1.04-1.23) | 0.003 |  | | 1.23 | | (0.97-1.54) | 0.08 |  |
|  | Cardiac disease | |  | 1.35 | (1.14-1.60) | 0.001 |  | | 1.24 | | (0.82-1.88) | 0.32 |  |
|  | Kidney dysfunction | |  | 1.75 | (1.10-2.79) | 0.02 |  | | 3.29 | | (1.46-7.42) | 0.004 |  |
|  | Cerebrovascular disease | |  | 1.28 | (1.05-1.56) | 0.02 |  | | 1.84 | | (1.19-2.84) | 0.01 |  |
|  | COPD | |  | 1.23 | (1.02-1.49) | 0.03 |  | | 1.00 | | (0.58-1.70) | 0.99 |  |
| CD, Clavien-Dindo classification; OR, odds ratio; CI, confidence interval; GPS, Glasgow prognostic score; ASA-PS, American Society of Anesthesiologists - Physical Status; cT, preoperative diagnosis of tumor invasion depth; cN, preoperative diagnosis of lymph node metastasis; COPD, chronic obstructive pulmonary disease. | | | | | | | | | | | | |  |
