# Supplementary material for: Significance of the Glasgow prognostic score for short‐term surgical outcomes: A nationwide survey using the Japanese National Clinical Database
Source: Ann Gastroenterol Surg. 2021 Mar 21;5(5):659–68. doi: 10.1002/ags3.12456 (PMC8452482; doi:10.1002/ags3.12456)
Supplement: Supplementary file 15 — Table S15 [file AGS3-5-659-s013.docx]

| **Table S15.** Frequent Postoperative Complications in Each Procedure | | | | |
| --- | --- | --- | --- | --- |
|  | **Postoperative complication** | **Number** | **%** |  |
| Eso | Pneumonia | 2802 | 13.6% |  |
|  | Anastomotic leakage | 2777 | 13.5% |  |
|  | Ogan/spase SSI | 1746 | 8.5% |  |
|  | Superficial incisional SSI | 1404 | 6.8% |  |
|  | Systemic sepsis | 714 | 3.5% |  |
| TG | Anastomotic leakage | 2008 | 4.8% |  |
|  | Ogan/spase SSI | 1996 | 4.8% |  |
|  | Pancreatic fistula | 1903 | 4.6% |  |
|  | Pneumonia | 1538 | 3.7% |  |
|  | Superficial incisional SSI | 949 | 2.3% |  |
| DG | Pancreatic fistula | 2865 | 2.6% |  |
|  | Ogan/spase SSI | 2836 | 2.6% |  |
|  | Anastomotic leakage | 2491 | 2.3% |  |
|  | Pneumonia | 2486 | 2.3% |  |
|  | Superficial incisional SSI | 1959 | 1.8% |  |
| RHC | Superficial incisional SSI | 2530 | 4.3% |  |
|  | Ogan/spase SSI | 818 | 1.4% |  |
|  | Anastomotic leakage | 793 | 1.4% |  |
|  | Pneumonia | 674 | 1.2% |  |
|  | Deep incisional SSI | 560 | 1.0% |  |
| LAR | Anastomotic leakage | 5873 | 9.4% |  |
|  | Ogan/spase SSI | 3742 | 6.0% |  |
|  | Superficial incisional SSI | 1830 | 2.9% |  |
|  | Systemic sepsis | 1039 | 1.7% |  |
|  | Deep incisional SSI | 628 | 1.0% |  |
| PD | Pancreatic fistula | 2203 | 11.0% |  |
|  | Ogan/spase SSI | 1499 | 7.5% |  |
|  | Anastomotic leakage | 1292 | 6.5% |  |
|  | Superficial incisional SSI | 916 | 4.6% |  |
|  | Bile leakage | 570 | 2.9% |  |
| Eso, esophagectomy; TG, total gastrectomy; DG, distal gastrectomy; RHC, right hemicolectomy; LAR, low anterior resection; PD, pancreaticoduodenectomy; SSI, surgical site infection. | | | | |
